# Deploying Wireless Sensor Networks in Multi-Story Buildings toward Internet of Things-Based Intelligent Environments: An Empirical Study

**DOI:** 10.3390/s24113415

**Published:** 2024-05-25

**Authors:** Nurul I. Sarkar, Sonia Gul

**Affiliations:** 1 Computer Science and Software Engineering, Auckland University of Technology, Auckland 1010, New Zealand; 2 Department of Computer Networks and Communications, College of Computer Sciences and Information Technology, King Faisal University, Al-Ahsa 31982, Saudi Arabia

**Keywords:** wireless sensor network (WSN), intelligent buildings, IoT

## Abstract

With the growing integration of the Internet of Things in smart buildings, it is crucial to ensure the precise implementation and operation of wireless sensor networks (WSNs). This paper aims to study the implementation aspect of WSNs in a commercial multi-story building, specifically addressing the difficulty of dealing with the variable environmental conditions on each floor. This research addresses the disparity between simulated situations and actual deployments, offering valuable insights into the potential to significantly improve the efficiency and responsiveness of building management systems. We obtain real-time sensor data to analyze and evaluate the system’s performance. Our investigation is grounded in the growing importance of incorporating WSNs into buildings to create intelligent environments. We provide an in-depth analysis for scrutinizing the disparities and commonalities between the datasets obtained from real-world deployments and simulation. The results obtained show the significance of accurate simulation models for reliable data representation, providing a roadmap for further developments in the integration of WSNs into intelligent building scenarios. This research’s findings highlight the potential for optimizing living and working conditions based on the real-time monitoring of critical environmental parameters. This includes insights into temperature, humidity, and light intensity, offering opportunities for enhanced comfort and efficiency in intelligent environments.

## 1. Introduction

In the contemporary technological landscape, the integration of wireless sensor networks (WSNs) into buildings represents a pivotal and transformative advancement [1]. This phenomenon is emblematic of the broader paradigm shift instigated by the Internet of Things (IoT), a revolution that has permeated nearly every facet of modern life. The advent of smart homes, smart buildings, and smart cities exemplifies this interconnected future, where the seamless exchange of information is orchestrated by a myriad of sensors strategically positioned within our living and working spaces. We used AS-XM1000 sensor networks for real-time monitoring of crucial environmental parameters such as temperature, humidity, and light intensity within the complex milieu of a multi-story commercial building environment.

Wireless sensor networks (WSNs) are increasingly utilized across various domains due to their ability to provide real-time monitoring and data collection. In addition to monitoring ambient properties such as humidity, light intensity, and internal temperature, WSNs play a pivotal role in civil engineering for structural health monitoring (SHM). In SHM, WSNs are employed to assess the integrity and safety of structures by monitoring parameters such as strain, vibration, and displacement. The wide range of applications underscores the versatility and importance of WSNs in both environmental monitoring and infrastructure management.

The present day is marked by the relentless evolution of technology, fostering a proliferation of interconnected devices. The IoT, as a driving force, has redefined the way we interact with our surroundings. It has created a web of interconnectivity, where devices communicate and collaborate to enhance efficiency, convenience, and overall user experience. The tangible manifestations of this interconnected future are evident in the emergence of smart homes, where devices seamlessly communicate to create an intelligent and responsive living environment. Smart buildings extend this concept to larger scales, incorporating sophisticated sensor networks to optimize energy usage, enhance security, and improve overall operational efficiency [2,3,4]. Furthermore, the vision of smart cities leverages IoT technologies to create urban spaces that are interconnected, efficient, and responsive to the needs of their inhabitants.

Building management system optimization in the era of smart buildings and IoT-enabled settings depends on the establishment of strong and dependable WSNs. The necessity of handling the difficulties caused by environmental unpredictability in multi-story structures, which can affect sensor network performance, is what spurred this research. Through the comparison of simulated results with real-world sensor data, this study sought to improve WSN accuracy and dependability, enabling more flexible and effective building operations. It is anticipated that the results will offer critical insights that may result in more thoughtful, adaptable, and sustainable building settings.

In this study, we used XM1000 sensors for deploying WSNs in commercial buildings. These sensors have programmable features and offer integrated wireless sensors within the framework of a WSN for capturing real-time data related to temperature, humidity, and light intensity. The choice of environmental parameters underscores their significance in understanding and optimizing living and working conditions. Temperature and humidity are pivotal factors influencing human comfort and health, while light intensity impacts productivity and the overall ambiance of a space.

The deployment strategy within a multi-story building environment adds complexity to this research, mirroring the real-world challenges associated with diverse and dynamic architectural settings. The multi-level approach aims to capture the nuances of environmental variations across different floors, providing a comprehensive understanding of how these parameters fluctuate within the built environment. By utilizing the Contiki operating system (OS) and a specialized simulator, this research incorporated a robust methodology to generate simulated data for comparison with real-world observations. This comparative analysis aimed to unravel the intricacies of sensor data accuracy in both simulated and real scenarios, contributing to the broader discourse on the reliability and applicability of simulation models in the field of WSNs.

This research, therefore, explored the potential of sensors within the context of WSNs in buildings, shedding light on their practical applications, challenges, and the broader implications for the evolution of smart environments in the contemporary era of IoT-driven advancements.

### 1.1. Research Challenges

In this paper, we address the following three research questions/challenges:

- Research Question 1: What sensor networks can be deployed for real-time monitoring of environmental parameters in multi-story buildings?

To address Research Question 1, we strategically deployed XM1000 sensors within a WSN in a multi-story building. Using the Contiki simulator, the sensors were programmed to monitor real-time data, focusing on critical environmental parameters like temperature, humidity, and light intensity. By adjusting the sampling interval to every five seconds, this study captured detailed environmental variations across different floors. The Contiki simulator was employed to generate simulated data, enabling a comparative analysis with real-world data. The findings not only contribute to a nuanced understanding of the deployment of sensors but also highlight implications for optimizing living and working conditions. This systematic approach, combining real-world monitoring, simulation, and comparative analysis, provides valuable insights into the practical applications of WSNs in complex building environments.

- Research Question 2: What insights can be gained from the comparative analysis of real-world sensor data and simulated data generated through the Contiki simulator, and how does this analysis inform our understanding of the reliability and accuracy of sensor networks in diverse architectural settings?

To address Research Question 2, we conducted a comparative analysis between real-world data collected by XM1000 sensors and simulated data. This approach involved utilizing the specialized Contiki simulator to emulate sensor behavior in controlled environments. Our analysis aimed to obtain insights into the reliability and accuracy of sensor networks, particularly in diverse architectural settings. By comparing actual sensor data with simulated results, our research sheds light on the alignment or disparities between the two datasets. This comparative analysis informs a nuanced understanding of how well sensor networks perform in real-world scenarios, thereby contributing valuable insights for enhancing the reliability and applicability of sensor networks in a variety of architectural environments.

- Research Question 3: In the context of the Internet of Things (IoT) and the evolution toward smart homes, smart buildings, and smart cities, how can the findings from this research on sensors be applied to enhance environmental surveillance, energy conservation, and overall building performance in intelligent environments?

To address Research Question 3, we investigated the application of sensors within the broader framework of the IoT and the development of smart homes, smart buildings, and smart cities. Through real-world monitoring and simulated data generation, this study provides insights into the deployment of XM1000 sensors in intelligent environments. The findings contribute to enhancing environmental surveillance, energy conservation, and overall building performance by offering a comprehensive understanding of how these sensors operate in diverse architectural settings. This study’s implications extend to optimizing living and working conditions, with the potential to inform the development of IoT-driven technologies, providing tangible benefits for environmental sustainability and building efficiency in the evolving landscape of intelligent environments.

### 1.2. Study Contribution

This paper’s main contribution is the creation and verification of a workable framework for installing wireless sensor networks (WSNs) in multi-story commercial buildings, improving Internet of Things (IoT)-based smart environments. This research offers significant perspectives on the integration of XM1000 sensors in WSNs and the intricate comprehension of these sensors’ operations in diverse architectural configurations. By establishing a comparison between simulated scenarios and real-world data, the Contiki operating system facilitates the assessment of the accuracy and dependability of sensor networks in intricate contexts. This makes a substantial contribution to the real-world uses of WSNs in intelligent building management, especially when it comes to improving environmental monitoring, energy efficiency, and overall building performance.

The main contributions of this paper are summarized as follows:

- We provide an analysis and valuable insights into the deployment of XM1000 sensors within a WSN in multi-story buildings, offering a detailed understanding of how these sensors function in diverse architectural settings.
- We developed a framework for the analysis of real-world data collected by sensors with simulated data generated through the Contiki simulator. This framework contributes to assessing the reliability and accuracy of sensor networks in complex environments.
- In the context of the IoT, we explored the practical applications of sensors for enhancing environmental surveillance, energy conservation, and overall building performance in smart homes, smart buildings, and smart cities.
- This study establishes a foundational contribution to the development of IoT-driven technologies, adding to the ongoing discourse on intelligent residences and structures. It underscores the significance of wireless sensor networks in advancing the capabilities of smart homes and buildings.

### 1.3. Structure of the Article

The rest of this paper is organized as follows: The related work on the deployment of WSNs is presented in Section 2. The research method adopted in this paper is discussed in Section 3. The sensor network deployment scenarios and results are discussed in Section 4. The simulation results are presented in Section 5. This paper concludes with Section 6.

## 2. Related Work

The emergence of WSNs has greatly advanced the progress of intelligent settings, utilizing the capacity to observe and manage many factors such as temperature, humidity, and light intensity. Research has focused on integrating WSNs into smart buildings and cities to improve efficiency, comfort, and sustainability.

The study conducted by Moreno M.V. et al. [5] investigated the use of sensor networks in intelligent buildings, with a particular focus on optimizing energy consumption and enhancing the comfort of occupants. Their research emphasized the significance of strategically positioning sensors and the function of adaptive algorithms in optimizing environmental conditions. In [6], Nguyen H.A. et al. expanded upon this discourse by investigating the application of WSNs in urban planning, with a particular focus on monitoring air quality. Their research demonstrated the adaptability of sensor networks in enhancing the intelligence and responsiveness of cities.

Simulation models have been developed in response to the complexity of real-world deployments in WSNs. In [7], Nayyar A. and R. Singh conducted a thorough examination of simulation tools utilized in WSNs, including NS-2. Their primary focus was on the precise modeling capabilities of these tools in precisely representing network protocols and sensor behavior. Afloogee [8] developed a sophisticated simulation framework that integrates environmental variables, thereby improving the accuracy of predicting sensor performance under various scenarios.

Environmental monitoring has experienced notable progress through the utilization of WSNs. Salaria A. et al. [9] examined the implementation of WSNs for the purpose of detecting forest fires. They emphasized the significance of utilizing temperature and humidity sensors to forecast the occurrence of fire incidents. Along a similar vein, ref. [10] investigated the application of sensor networks in agricultural environments for the purpose of monitoring soil moisture. Their study showcased the promising capabilities of WSNs in the field of precision agriculture.

Despite the advancements, there are still obstacles in the implementation and simulation of WSNs. Dogra R. et al. [11] identified the challenges related to the scalability of sensor networks and their energy consumption. They proposed that future research should focus on developing energy-efficient sensor designs and network protocols. In [12], Said O. and A. Tolba stressed the need for more advanced simulation models that can effectively replicate the intricacies of the actual world. They proposed the integration of AI and machine learning approaches to enhance the accuracy of simulation results.

The existing literature emphasizes the crucial importance of WSNs in the advancement of smart environments, particularly in environmental monitoring. Simulations provide a potent means of comprehending the behaviors of sensor networks. However, a persistent issue lies in bridging the disparity between simulated and real data. Subsequent investigations should focus on resolving these inconsistencies by investigating novel methodologies to improve the precision, dependability, and relevance of WSNs in intelligent settings.

Ongoing research is investigating the effectiveness of wireless sensor networks (WSNs) in optimizing energy usage and monitoring the environment intelligently. The study conducted in [13] introduced a sophisticated energy management system for intelligent buildings. This system utilizes sensor data to adaptively regulate energy usage in response to occupancy and usage patterns, resulting in a substantial reduction in energy wastage. A paper [14] presents an enhanced simulation framework for WSNs in the context of simulation tools. This framework takes into consideration real-time environmental changes and adjusts sensor operations, accordingly resulting in a more precise representation of sensor performance under different conditions.

Another study [15] contributed to the advancement of deploying WSNs in urban areas. This research showcases the potential of WSNs in traffic management systems for smart cities. By analyzing real-time data, WSNs can optimize traffic flow and alleviate congestion. Moreover, in [16], the authors emphasize the practical use of WSNs in agricultural environments. This study utilized soil and climate sensors to provide information to irrigation systems, resulting in water conservation and improved crop yield.

These studies have enhanced the current body of knowledge by offering practical frameworks for the implementation and assessment of WSNs in intelligent environments. They have emphasized the significance of precise simulation models and real-world testing.

The summary of the related work on WSN deployment scenarios is presented in Table 1. The main research contribution and year of publication are listed in Columns 3 and 2, respectively. For each main contribution, we examined the aspects of sensor deployment, simulation performance study, IoT-based implementation, and system deployment cost. The sensor deployment, simulation study, IoT-based implementation, and system deployment cost are listed in Columns 4–7, respectively.

## 3. Methods: System Design and Analysis

The aim of the research methodology employed in this study was to offer a thorough comprehension of the system design and analysis of sensor network deployment within a multi-story building setting. Field trial sensor data measurement was one of the main research methods adopted in this study. The process included gathering device information, developing a network model, collecting sensor data, system simulation, and comparing real data with simulated data. The objective was to utilize a methodical strategy that integrates actual experimentation with simulation-based analysis to attain resilient and dependable outcomes.

**Figure 1:**
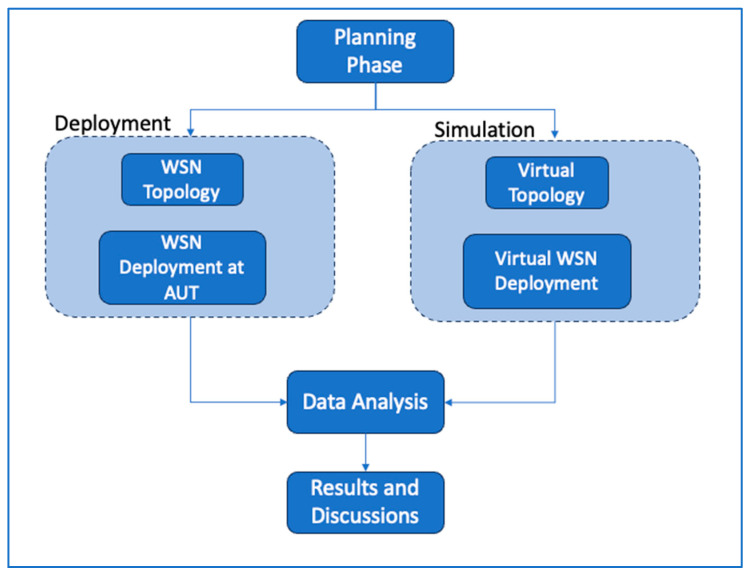
Research method adopted.

The establishment of a WSN in the building context was a crucial element of this phase, with an emphasis on creating an environment that accurately replicates real-world situations. Figure 1 shows the research design and methodology employed for conducting this study. The adopted methodology is appropriate for investigation, especially system deployment and simulation study within a multi-floor university campus building settings. The following detailed steps outline the approach taken in each phase.

Our research began with a planning phase that aimed to explicitly define the research objectives, with a particular focus on the critical environmental elements of temperature, humidity, and light intensity. The multi-story building at Auckland University of Technology (AUT) was selected for sensor deployment and testbed measurements due to its availability and appropriateness for our study. Following the design stage, this study split into two parallel directions: (i) sensor network deployment and (ii) system simulation.

The WSN topology was carefully designed during the deployment phase. This meant designing the WSN architecture while accounting for the intricacies of spatial configuration, connectivity requirements, and potential disruptions. AS-XM1000 sensor nodes were deployed on two multi-story university buildings. More on building layouts and measurement locations is provided in Section 4.

### 3.1. Hardware and Software Setup for Investigation

**Figure 2:**
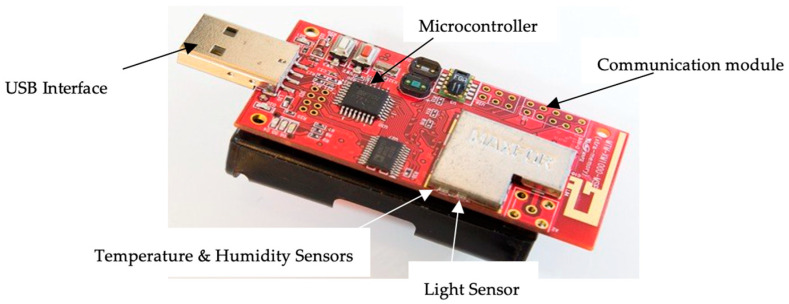
Wireless sensor node (AS-XM1000) used in system deployment.

We developed a virtual client–server topology using the Contiki 2.7 simulator (Ubuntu operating system) for a system performance study. The simulator was configured to precisely mimic the physical attributes of the university’s WA Library building. Additionally, a virtual representation of the WSN with preset node locations and communication channels was created. We programed the AS-XM1000 sensor nodes to generate simulated data for temperature, humidity, and light intensity for the virtual WSN deployment. The Contiki simulator (http://www.contiki-os.org/start.html, accessed on 20 April 2024) was used to generate simulated data, ensuring adherence to the previously defined virtual WSN architecture. In Figure 2, two sensors are labeled separately. One sensor was for temperature and humidity, and the other one was a light sensor.

The initial stage of the study entailed a thorough investigation of the AS-XM1000 hardware platform. This involved comprehending the technical specifications of the device, its integration with Contiki, and the functions of the embedded sensors (temperature, humidity, and light intensity). The objective was to conduct the groundwork for the eventual installation and programming of the devices in the WSN.

### 3.2. Sensor Data Collection and Analysis

**Figure 3:**
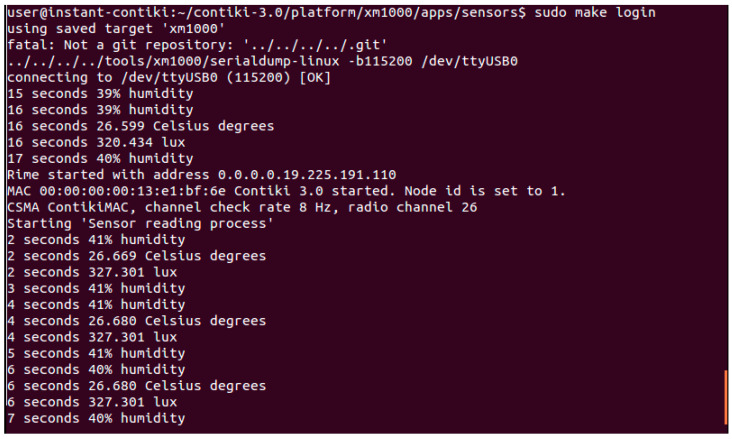
WSN configuration setup for sensor data collection and validation.

A variety of sources including simulated and real-world data were gathered and combined to create a large amount of dataset. Real and simulated data were gathered methodically. The dataset was thoroughly analyzed to find patterns, similarities, and differences. The findings were consolidated at the “Results and Discussions” stage. A thorough summary of the findings and observations from the deployment and simulation phases are discussed in this paper. The significance of the findings for the dependability of sensor networks and their uses in intelligent environments was carefully considered while analyzing the ramifications of the findings. This creates a solid foundation for deriving insightful data and recommendations pertinent to upcoming sensor network deployments and simulations. Figure 3 shows a screenshot of the WSN configuration for sensor data collection.

### 3.3. System Deployment Scenarios

*Network Model:* After obtaining the device’s information, we created network models. This encompassed the systematic creation of the experimental setting, which entailed the installation of XM1000 platforms, uploading of applications, and programming of devices to obtain real-time sensor data.

In the deployment phase, we further explored the real-world application of the WSN AUT WT Tower building, utilizing XM1000 sensors. It offered a thorough explanation of the implementation’s several facets.

**Figure 4:**
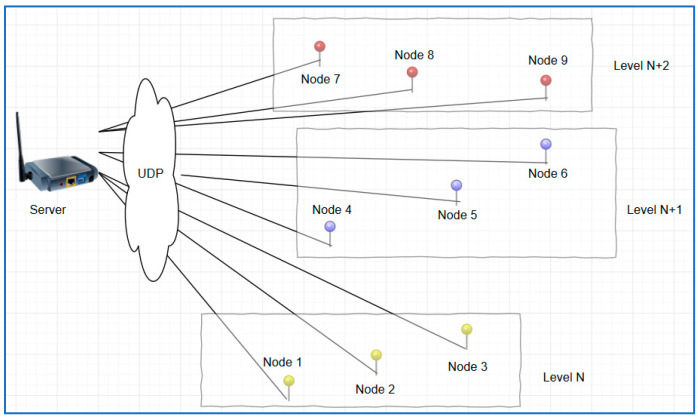
The proposed WSN deployment architecture.

Figure 4 shows the network topology employed in this investigation, which also visually displays the placement of sensors on three floors of the WT tower building. Three to four XM1000 platforms were placed strategically on each level to guarantee the best possible data collection.

A basic UDP connection was established between two XM1000 modules. One module functioned as the server, while the other functioned as a node, demonstrating the essential communication framework that underlaid the complete WSN. This study was conducted using three topological scenarios, as discussed below.

#### 3.3.1. Scenario 1: Topology of a Single Server and Client

**Figure 5:**
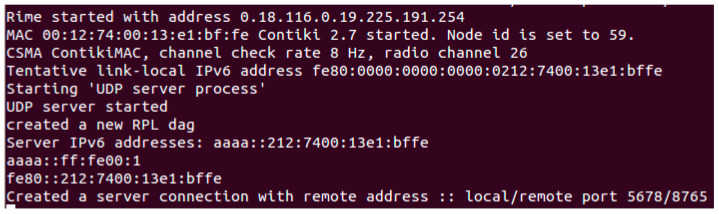
Scenario 1 (single server and a client).

In this configuration (Scenario 1), we had one client and one server. The client sensor was battery-powered for flexible deployment within its coverage area. The server was physically connected to a laptop. Scenario 1 offered a thorough investigation of the deployment of WSN. Communication and message exchange took place between the server and client, emphasizing the vital role that UDP plays in making this connection possible. The Contiki scripts were responsible for setting the server’s node ID, IP address, port number, and MAC address. Figure 5 shows a screenshot of server’s information when everything is operating as it should.

#### 3.3.2. Scenario 2: Configuration of One Server and Two Clients

**Figure 6:**
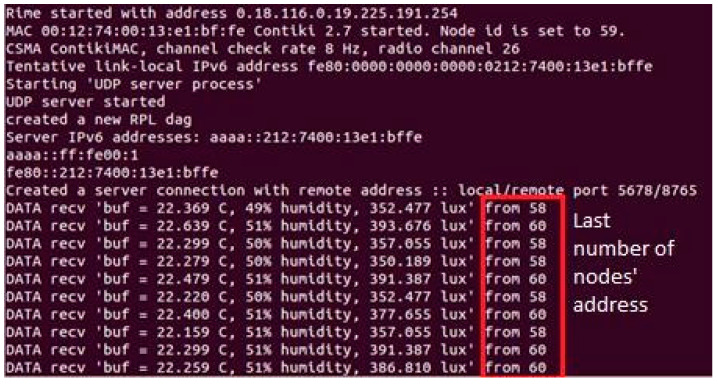
Scenario 2 (one server and two clients).

In this configuration (Scenario 2), we had two clients and one server located at a specific floor level in the WA Library building. Scenario 2 broadened the configuration of Scenario 1, as discussed earlier. The system provided a thorough understanding of the surrounding conditions by presenting data readings of temperature, humidity, and light intensity from various points on the same floor. Figure 6 shows the screenshot server’s information for Scenario 2.

#### 3.3.3. Scenario 3: Topology of a Single Server and Multiple Clients

In this configuration (Scenario 3), we had multiple clients and one server. We deployed sensors on three floors of the WT Tower building. Nine sensors were deployed; unique node IDs and addresses were assigned to each sensor. These sensors were battery-operated. The deployment strategy entailed sending real-time data to the server connected to the laptop at regular intervals once it had been systematically collected. It should be noted that the sensors were equipped with adequate battery capacity, which rendered battery replacements unnecessary throughout the experiments. The research findings are presented next.

## 4. Results and Discussion

This section explores the practical aspects of data collection within the WA Tower, specifically focusing on the early obstacles encountered and the subsequent decisions made. For data collection and analysis purposes, we divided our experiments into multiple plans.

### 4.1. Study 1: Deployment of Sensor Nodes on Floor 6 of WA Library Building

**Figure 7:**
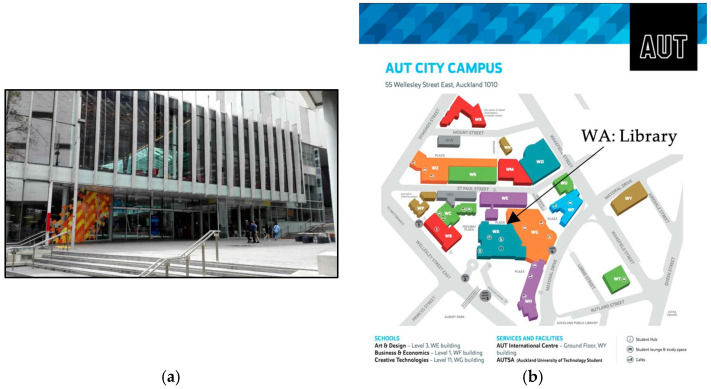
(**a**) External view of university WA library building; and (**b**) AUT city campus map (https://www.aut.ac.nz/__data/assets/pdf_file/0011/118919/AUT-campus-map-city.pdf, accessed on 20 April 2024).

**Figure 8:**
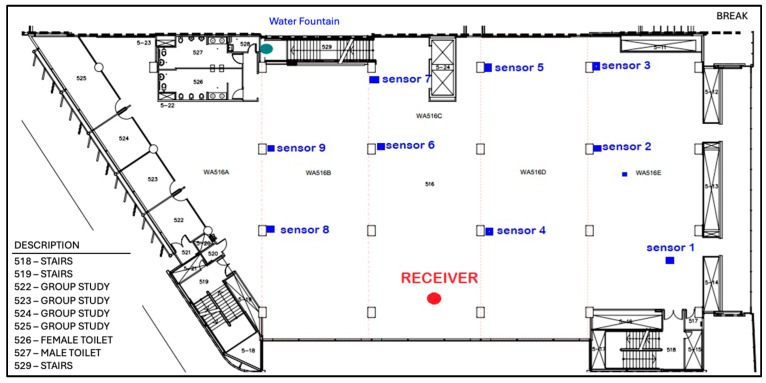
Sensor node deployment on Floor 6 of WA library building.

Figure 7 shows the physical layout of Auckland University of Technology (AUT) WA building. Figure 8 shows the deployment of wireless sensors nodes on Floor 6 of the WA building. We obtained sensor data with a particular focus on important factors such as temperature, humidity, and light intensity. Analysis was a crucial element in assessing the effectiveness, dependability, and performance of the deployed WSN, offering useful insights for future discussion and interpretation of the results.

**Figure 9:**
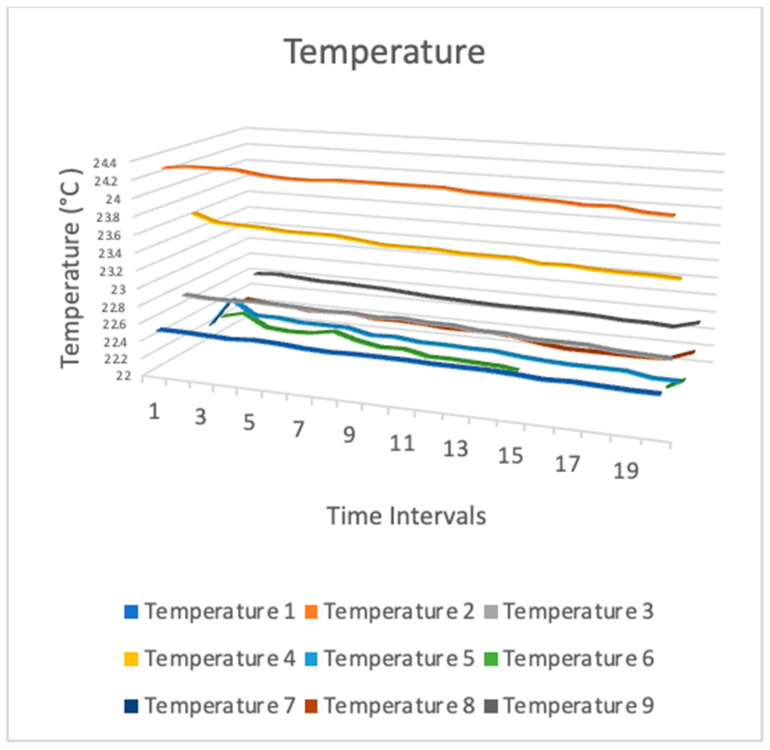
Temperature sensor data measurement (3-dimension line chart).

**Figure 10:**
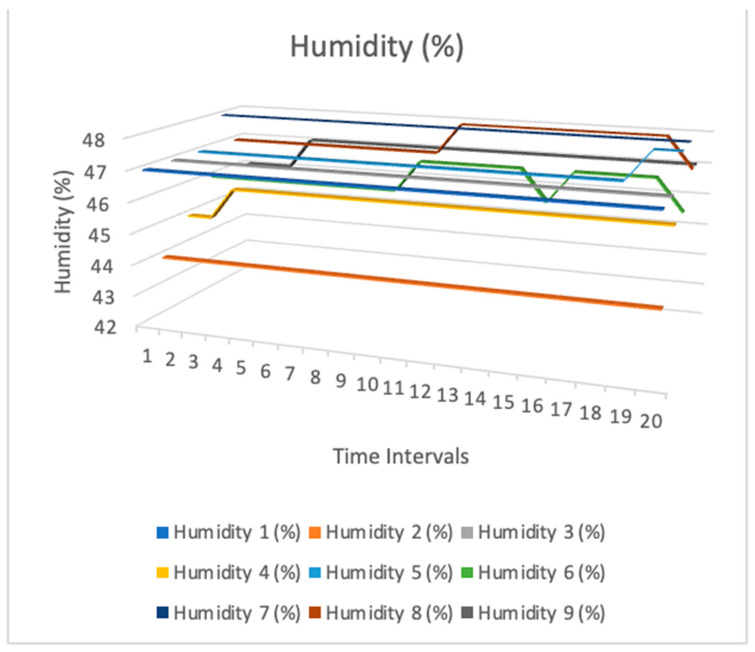
Humidity sensor measurement data.

**Figure 11:**
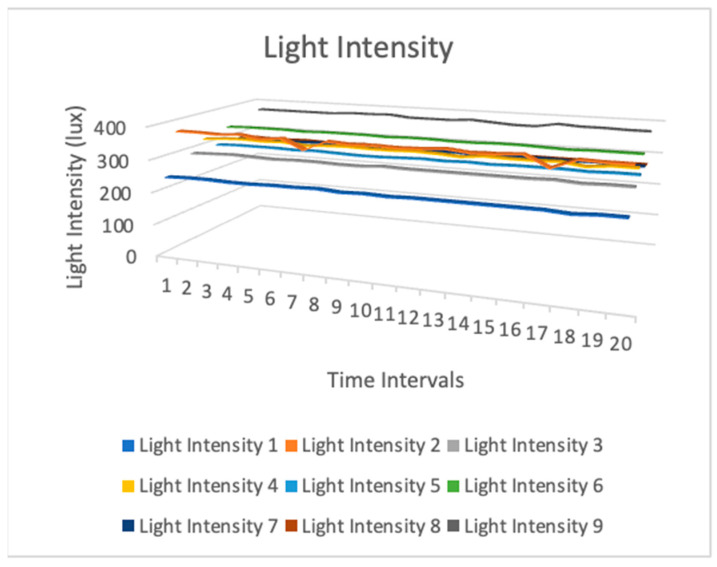
Light intensity sensor measurement data.

Figure 9, Figure 10 and Figure 11 show temperature, humidity, and light intensity trends. The three-line charts use the same data. One chart compares temperature, humidity, and light intensity.

Figure 9 shows various temperatures observed on the sixth floor of the University WA building (library building). In the graph, the x axis represents the sequential time intervals at which sensor data were recorded, while the y axis denotes the temperature measured in degrees Celsius. Nine sensors were placed in nine bookcases on the sixth floor of the library building. Even on the same floor, the temperature of various bookshelves was discernible. Various factors such as the presence of students and the amount of sunshine might have influenced the temperature. This demonstrates the correlation between the temperatures recorded by these sensors, indicating an examination of the spatial dispersion of heat or the reliability of the sensors’ measurements.

Figure 10 establishes a connection between the data collected by these sensors and the levels of humidity, suggesting that each sensor was capable of measuring both temperature and humidity. Understanding the interaction between these two environmental variables in the research area is of utmost importance. Figure 10 shows that the humidity levels were not uniform, and air flow may have been one of the reasons. The airflow had the potential to distribute moisture throughout all areas. This process may have led to varying levels of humidity in various bookcases.

Figure 11 shows the association between the light intensity levels measured using the nine sensors. It shows the spatial distribution of light intensity across various sites or the level of agreement between readings from multiple sensors. This figure illustrates the luminosity surrounding the nine bookshelves. It was also unique and dynamic. Certain areas surrounding the bookshelves were illuminated, such as those exposed to sunlight or those near light bulbs. Some were situated in obscure or dimly lit areas. Consequently, the levels of light intensity varied.

### 4.2. Study 2: Deployment of Sensor Nodes on Floors 3 and 4 of WT Tower Building

**Figure 12:**
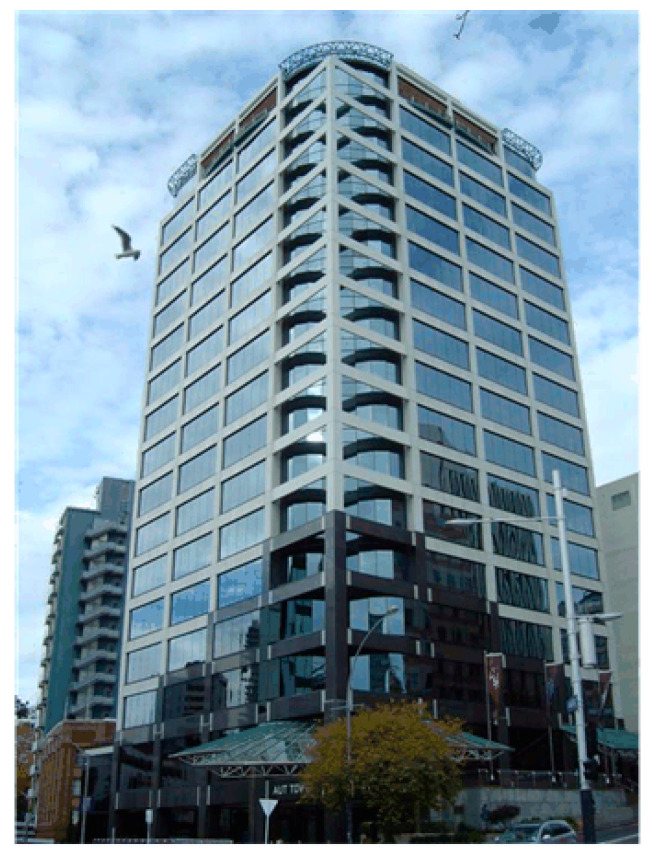
External view of WT tower building.

**Figure 13:**
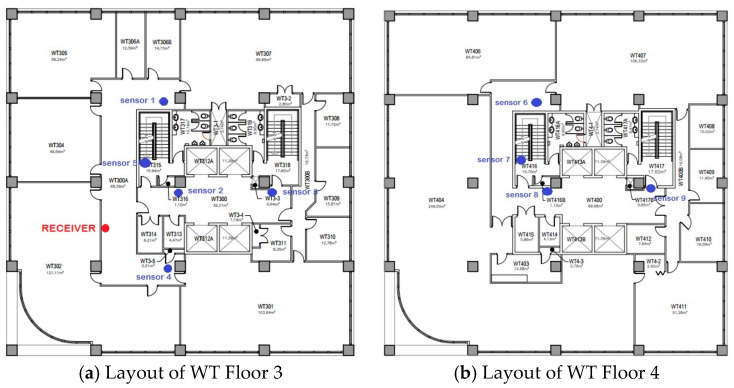
Deployment of sensor nodes on Floors 3 and 4 of WT tower building.

Study 2 dealt with the sensor network deployment on the third and fourth floors of the WT tower building. Figure 12 shows an external view of WT tower building. The layouts of WT Floors 3 and 4 are shown in Figure 13a,b, respectively. A total of five sensors were deployed on Floor 3, and four sensors were deployed on Floor 4.

Additionally, one receiver node was deployed on Floor 3 to collect sensor data. Sensors 1 to 6 were placed on Floor 3, while sensors 7 to 9 were placed on Floor 4. The vertical distance between two floors was relatively shorter compared to that of the WA library building. Therefore, despite the sensors being located at different elevations, the signal possessed sufficient strength to reach the server located on a separate floor.

**Figure 14:**
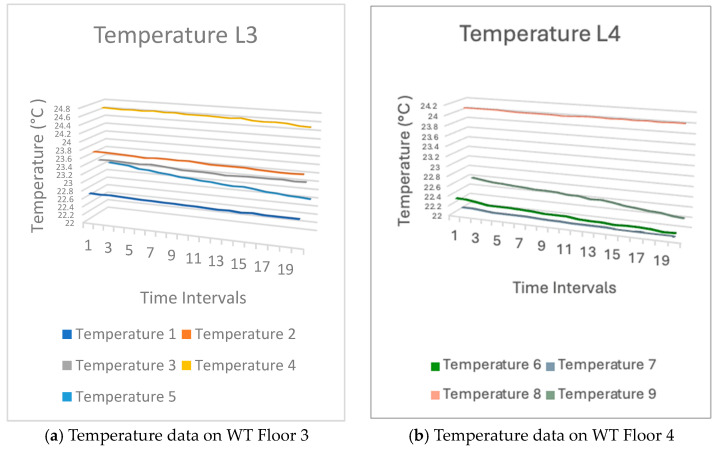
Temperature sensor data from WT tower building.

Upon analyzing the sensor data, we found that the temperature on Floor 3 was approximately 23.5 degrees Celsius and the temperature on Floor 4 about 22.5 degrees Celsius. The temperature varied based on the specific locations where the sensors were positioned on each floor. A set of sensors was strategically positioned adjacent to the entrance, resulting in an accelerated airflow and a decrease in temperature. A few were stationed within the room. Consequently, the temperature was elevated. Figure 14 shows the sensors temperature readings on both Floors 3 and 4.

**Figure 15:**
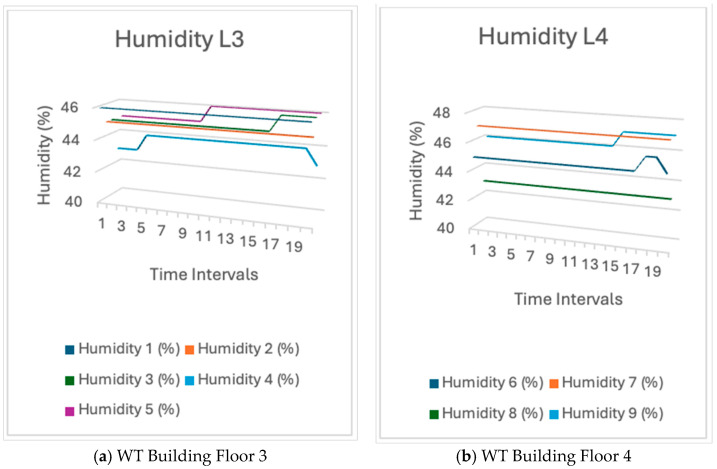
Humidity sensor measurement readings from sensors in WT building.

The humidity trend in the WT building was comparable to that in the WA building. The reason may be identical to that of temperature. The phenomenon is caused by air movement. Therefore, the moisture levels would vary and be subject to fluctuation. Figure 15 shows the sensor humidity readings.

**Figure 16:**
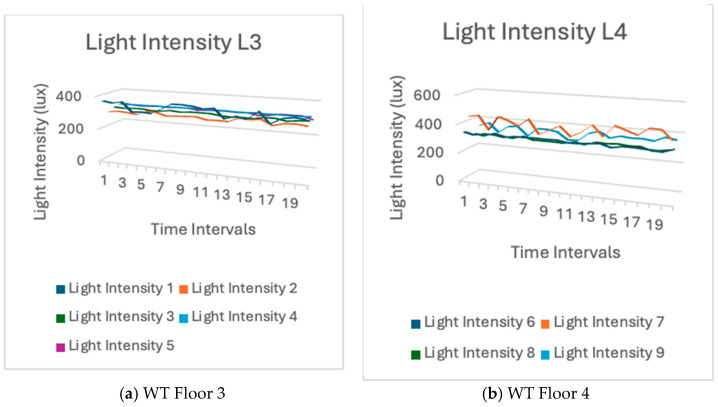
Light intensity measurement from sensor data in WT building.

The variation in light intensity on level four was more pronounced than that on level three. There were more individuals on level four than on level three. As they walked past the sensors, their shadows loomed over the sensors. The luminosity underwent a variation. As the number of individuals increased, an increasing number of shadows loomed. Therefore, the light intensity varied. This is evident from the sensors’ readings of light intensity. Figure 16 shows the light intensity variations in level three and level four of the WT building.

We verified the accuracy of our research findings by simulation. The following section provides an in-depth analysis of the simulation configuration and the outcomes we achieved.

## 5. System Simulation and Results

This section discusses the simulation environment, where we created multiple scenario plans and obtained appropriate results. Simulation results were generated for the variables of temperature, humidity, and light sensitivity. These results were then compared with the outcomes of our deployed network.

### 5.1. Simulation Environment and Setup

We used the Contiki simulator for the simulation of sensor networks. The process involved the installation of nodes, adding motes, and sending signals among the motes.

**Figure 17:**
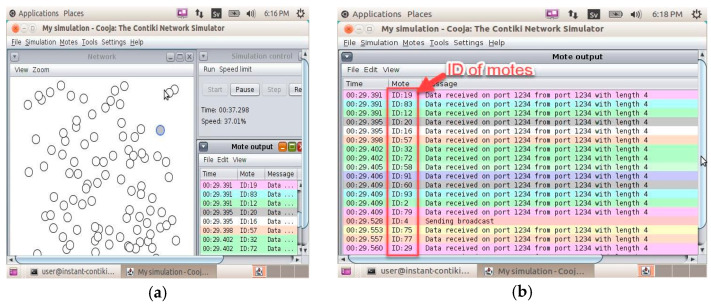

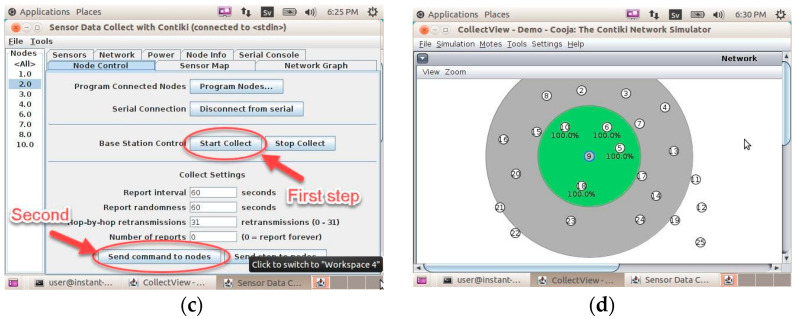
Simulation setup: (**a**) temperature; (**b**) humidity; (**c**) light intensity 1; and (**d**) light intensity 2.

Figure 17 shows the simulation setup used to measure the key parameters such as temperature, humidity, and light intensity using the Contiki Cooja 2.7 [17] simulator operating on operating system. In simulation environment, we created 25 motes (equivalent to 25 sensors) for client (sensor)-to-server connectivity.

**Figure 18:**
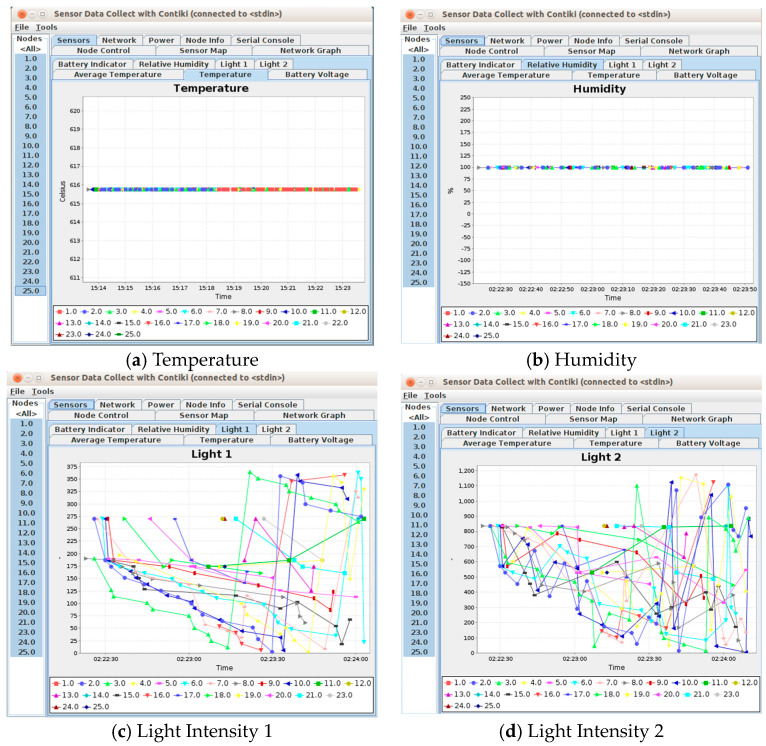
Simulation results: (**a**) temperature; (**b**) humidity; (**c**) light intensity 1; and (**d**) light intensity 2.

Figure 18 shows the simulation results in the form of a line chart. The simulator emulated 25 sensor nodes in a WSN within a building or a home. The findings indicated consistent levels of temperature and humidity, although the measurements of light intensity exhibited fluctuations.

The simulation results (Figure 18a) show that the temperature readings maintained a consistent level of uniformity. The consistent nature of the simulated temperature data can be attributed to the regulated settings within the simulation environment. In contrast to real-world environments, simulations can control and keep environmental elements constant, such as airflow, sun radiation, and heat sources, that would normally cause fluctuations in temperature. The same is true for humidity, as shown in Figure 18b.

However, Figure 18c,d indicate fluctuations in the light intensity. The observed variations in the light sensitivity results in the simulation can be ascribed to the dynamic characteristics of light levels in an environment, which might undergo frequent and rapid changes due to many variables. The model is meant to accurately represent variations in light circumstances, including the shift from day to night, the influence of artificial lighting, and changes in natural light caused by weather conditions. These variations can lead to oscillations in the measured intensity of light.

### 5.2. Result Validation and Discussion

We compared the simulation results to the results obtained from our field trial (practical sensor network deployment scenarios). The details regarding the validation of the temperature, humidity, and light intensity results are discussed next.

- Temperature: When comparing the testbed and simulation results for temperature, there was a noticeable difference in the recorded values and how they were distributed. On Floor 3, the average temperature in the actual setting was around 23.5 degrees Celsius, slightly surpassing the average temperature of roughly 22.5 degrees Celsius on Floor 3. The discrepancy in temperature measurements obtained from the actual surroundings could be traced to the specific positions where the sensors were placed. For instance, sensors positioned near the door, where air circulation was more rapid, detected lower temperatures, whereas those situated within a room exhibited greater temperatures because of the limited air movement.

In contrast, the simulated data exhibited consistency with the temperature measurements among the sensors, although the values differed from those of the actual data. The simulation yielded temperature values that were markedly lower than those seen in the actual environment. The actual sensor data results had an average temperature of around 23 degrees Celsius; however, the simulated temperature data constantly remained below 43 degrees Celsius.

The disparity between the test and simulation outcomes may have arisen from the constraints on the simulation environment. The simulation did not consider all the intricate variables that influence temperature, such as the air currents in proximity to doors, insulation characteristics of the rooms, heat discharges from equipment or individuals, and other microclimate conditions within the building. Thus, although the simulated temperature data remained constant, like the generally stable real-world data, they failed to replicate the true values and subtle changes that existed in the real environment.

- Humidity: The real-world test data suggested that the average humidity on level three and level four was influenced by air movement, demonstrating that environmental influences had an impact on the humidity levels reported by the sensors. Moreover, the humidity levels may have varied due to factors such as ventilation and the presence of apertures such as doors and windows, which facilitate air circulation. Conversely, the software’s simulation findings demonstrated a consistent humidity level. The overall stability in the simulation matched the real-world data, with an average humidity fluctuating between 43% and 47%.
- Light Intensity: The test results revealed changes in light intensity, notably on level four, which were more pronounced in comparison to those on level three. The increase in foot traffic on level four was responsible for this phenomenon. As more individuals walked past the sensors, their shadows created momentary fluctuations in the reported light levels. The empirical data, thus, demonstrated a clear association between human activity and changes in light intensity. Similarly, the simulation results exhibited significant variations in light intensity.

### 5.3. Theoretical Analysis

The deployment of WSNs in multi-story buildings is theoretically supported by the requirements for the precise modeling of the environmental characteristics that have a significant impact on occupant comfort and energy usage. The examination of signal propagation through different building materials, the thermodynamic behavior of constructed environments, and the dynamic nature of environmental elements like temperature, humidity, and light intensity are all included in this.

In this work, we discussed the theoretical foundation for the actual implementation of XM1000 sensor deployment in a WSN. Our method carefully simulated the stochastic nature of environmental change, the physics of sensor signal attenuation over floors, and the temporal integrity of the sensor data. We created a virtual topology that replicated the physical characteristics of the deployment environment by using the Contiki operating system and its specific simulation capabilities. This enabled predictive analytics and the fine tuning of sensor placement for optimal data collection.

To address the difficulties of implementing WSNs in intricate architectural settings, our theoretical study highlights the necessity of a multidisciplinary approach that incorporates concepts from wireless communications, building science, and systems engineering. Our investigation delved into the theoretical ramifications of the gathered sensor data, with a particular emphasis on verifying simulation models through empirical measurements for intelligent building management systems.

This investigation also looked at how machine learning algorithms might be used to process and understand massive datasets from sensor networks. This could result in more efficient predictive modeling and adaptive control systems of smart building energy efficiency. The foundation for our proposed WSN deployment’s resilience was built in this part, paving the way for future developments in IoT applications for smart urban infrastructure.

### 5.4. Practical Implications

The practical implications of this study on the WSNs implemented in commercial buildings for IoT-based smart cities are extensive. Our research demonstrates that implementing WSNs is a feasible approach to enhancing building management. The results presented in this paper provide some insights into the most effective positioning of sensors and the capacity for immediate environmental monitoring, facilitating the flexible control of building conditions.

The results obtained can be useful for IoT-based smart homes, buildings, and even smart cities. Each home or building could have one server and multiple nodes. Nodes collect environmental data and send them to a server that analyzes data and compares threshold values and controls relative devices. For instance, if the temperature is lower than the threshold value, the server turns on s heater. If humidity is a bit high, the server switches on a drier. If it is dark in the room, the server turns on lights or open electric curtains. In the future, WSNs could be deployed in every home and building. Servers transmit data to the main computer in the city. The computer could send commands to every server in commercial buildings to realize integrated maintenance.

Implementing such a system has the potential to result in significant financial savings for building operators by optimizing resource utilization and enhancing environmental conditions, hence positively impacting the inhabitants’ well-being. These findings are crucial as they can provide valuable insights for future Internet of Things (IoT) deployments in intelligent buildings and cities, ultimately contributing to the overarching objective of establishing sustainable, efficient, and comfortable living and working environments.

### 5.5. High-Density Sensor Deployments—Issues and Challenges

The problems of range and potential collisions occur while implementing WSNs in multi-story structures, which are influenced by various crucial factors.

The sensor range is determined by its design and the climatic conditions of the structure. Obstacles like walls, floors, and ceilings might weaken signals, hence impacting the sensors’ capacity to establish communication with the network. When sensors are placed in various locations and architectural environments, the range of their transmission can vary, resulting in possible areas where sensor data may not be properly sent.

Signal collisions become more likely in surroundings with a large concentration of sensors. Collisions arise when numerous sensor nodes make simultaneous attempts to transmit data over the network, resulting in interference and the possibility of data loss. The probability of such occurrences increases in intricate deployments within multi-story structures where numerous sensors operate in proximity.

To address these problems, we suggest implementing advanced network protocols and engaging in careful planning. To decrease the risk of data collisions and range difficulties and ensure reliable data gathering and transmission inside the WSN, it is important to ensure that communication channels do not overlap, to implement efficient time-division multiplexing, and to utilize adaptive signal processing techniques. The document presented a systematic method for installing wireless sensor networks (WSNs) in such situations. It serves as a basis for tackling these difficulties through real implementation and simulation.

## 6. Conclusions and Future Directions

This study methodically investigated the deployment scenarios and emulation of WSNs in a multi-story commercial building setting, with a specific emphasis on temperature, humidity, and light intensity as crucial environmental factors. This paper outlined a thorough approach that includes the selection of devices, the building of a network model, the gathering of data, and the comparison between real-world and simulated data. The key findings emphasized the disparities and resemblances between the actual deployment outcomes and the results of the simulation.

We found that although the simulation successfully represented the overall consistency of temperature and humidity, it was unable to precisely reproduce the subtle fluctuations detected in the real-world data. The simulation results demonstrated a lack of accuracy in representing the spatial changes and airflow effects observed in the physical environment, particularly in terms of temperature and humidity homogeneity. Conversely, the simulated outcomes for light intensity exhibited substantial oscillations, reflecting the anticipated variability in a real-life environment. In this paper, we provided working knowledge on the deployment aspect of sensor networks in intelligent environments for smart buildings and cities. Developing a technique to improve system accuracy and dependability is suggested as future work.

In future research, we plan to deepen the comparative study between our XM1000-sensor-based WSN framework and existing energy optimization technologies. We will benchmark sensor performance, develop predictive energy management algorithms, and integrate with other IoT systems for comprehensive energy conservation assessments. Longitudinal studies and machine learning models will be utilized to predict and enhance energy efficiency while also evaluating the economic impacts. This will help with not only validating the effectiveness of our approach but also identifying enhancements to foster energy optimization in smart buildings.

## Figures and Tables

**Table 1:** Summary of the related work on WSN deployment scenarios.

Reference	Year	Main Contribution	Sensor-Deployment	Simulation	IoT-Based	Low-Cost
[5]	2014	Proposed a building automation platform that controls the actuators built into the system and gathers and monitors all data related to the issue of building energy consumption.	Yes	No	Yes	No
[7]	2015	This article reviews wireless sensor network simulation tools to help researchers choose the best one for simulating and testing in their study.	No	No	No	No
[13]	2018	Proposed a user-friendly, scalable IoT-based system that uses real-time sensor data to inform occupants of their energy consumption and provide personalized recommendations for energy savings and comfort optimization.	Yes	Yes	Yes	No
[15]	2020	Proposed a WSN-based intelligent traffic control system that uses IoT and mobile apps to notify drivers about traffic density and parking availability in smart cities to reduce congestion.	No	Yes	Yes	No
[10]	2021	Proposed a cost-effective wireless sensor network consisting of sensor nodes designed to measure soil moisture.	Yes	No	Yes	Yes
[11]	2021	Proposed a cluster-based routing mechanism that can be implemented in the sensing layer of smart-city IoT.	No	Yes	Yes	No
[6]	2022	Proposed a low-cost wireless sensor network that is enabled by the Internet of Things that greatly enhances the dependability of air quality monitoring in suburban regions.	No	Yes	Yes	Yes
[8]	2022	Proposed an IoT simulation framework for wireless sensor networks that will be used for monitoring the environment.	No	Yes	Yes	No
[9]	2022	Forest fire causes, damages, and impacts are covered in this study.	No	No	No	No
[14]	2024	Proposed advanced IoT and industrial IoT (IIoT) research and development by providing a diverse and realistic testing environment for smart city innovations and a variety of technologies.	Yes	Yes	Yes	No
**Our work**	Deployment of wireless sensor networks in commercial buildings towards IoT-based intelligent environments		Yes	Yes	Yes	Yes

## Data Availability

The original contributions presented in the study are included in the article, further inquiries can be directed to the corresponding authors.
